# Imiquimod enhances excitability of dorsal root ganglion neurons by inhibiting background (K_2P_) and voltage-gated (K_v_1.1 and K_v_1.2) potassium channels

**DOI:** 10.1186/1744-8069-8-2

**Published:** 2012-01-11

**Authors:** Jaekwang Lee, Taekeun Kim, Jinpyo Hong, Junsung Woo, Hyunjung Min, Eunmi Hwang, Sung Joong Lee, C Justin Lee

**Affiliations:** 1 WCI Center for Functional Connectomics, Korea Institute of Science and Technology, Seoul, 136-791, Republic of Korea; 2 Department of Neuroscience, Dental Research Institute, and Brain Korea 21, School of Dentistry, Seoul National University, Seoul, 110-749, Republic of Korea

## Abstract

**Background:**

Imiquimod (IQ) is known as an agonist of Toll-like receptor 7 (TLR7) and is widely used to treat various infectious skin diseases. However, it causes severe itching sensation as its side effect. The precise mechanism of how IQ causes itching sensation is unknown. A recent report suggested a molecular target of IQ as TLR7 expressed in dorsal root ganglion (DRG) neurons. However, we recently proposed a TLR7-independent mechanism, in which the activation of TLR7 is not required for the action of IQ in DRG neurons. To resolve this controversy regarding the involvement of TLR7 and to address the exact molecular identity of itching sensation by IQ, we investigated the possible molecular target of IQ in DRG neurons.

**Findings:**

When IQ was applied to DRG neurons, we observed an increase in action potential (AP) duration and membrane resistance both in wild type and TLR7-deficient mice. Based on these results, we tested whether the treatment of IQ has an effect on the activity of K^+ ^channels, K_v_1.1 and K_v_1.2 (voltage-gated K^+ ^channels) and TREK1 and TRAAK (K_2P _channels). IQ effectively reduced the currents mediated by both K^+ ^channels in a dose-dependent manner, acting as an antagonist at TREK1 and TRAAK and as a partial antagonist at K_v_1.1 and K_v_1.2.

**Conclusions:**

Our results demonstrate that IQ blocks the voltage-gated K^+ ^channels to increase AP duration and K_2P _channels to increase membrane resistance, which are critical for the membrane excitability of DRG neurons. Therefore, we propose that IQ enhances the excitability of DRG neurons by blocking multiple potassium channels and causing pruritus.

## Findings

Imiquimod (IQ) is widely used to treat various skin diseases such as molluscum contagiosum, basal cell carcinoma, and Bowen's disease [1]. Topical application of the compound is currently approved for treatment of genital warts, a highly contagious sexually transmitted disease caused by human papillomavirus [1-5]. It is believed that IQ modulates immune responses via Toll-like receptor 7 (TLR7) releasing IFN-α/β and proinflammatory cytokines [6]. Nevertheless, the exact mechanism of how IQ activates the immune system is not fully understood. Despite its clinical importance, the most prominent side effect of IQ is pruritus, e.g. itching sensation [7,8]. The mechanism of how IQ causes itching sensation has remained unknown.

Recently we and others reported that IQ-induced depolarization in dorsal root ganglion (DRG) neurons leads to itching behavior [7,8]. Even though these two studies agreed upon IQ's action on the cellular and behavioral level, there was a profound discrepancy on the molecular target of IQ. The study by Liu et al. reported that membrane depolarization caused by IQ required TLR7 to generate action potentials (APs) and to induce itching behavior [8]. On the contrary, our study independently demonstrated that the action of IQ does not require the TLR7 pathway [7]. In this study, we showed that IQ caused depolarization and AP firing in DRG neurons of both wild type (WT) and TLR7 knock out (KO) mice. Our results suggested that IQ induced depolarization and AP firing, not by TLR7 signal transduction, but by the direct effect on other channels [7].

DRG neurons express many types of potassium channels including voltage-gated (K_v_), inwardly rectifying (K_ir_), Ca^2+^-activated (K_Ca_) and background (leak, K_2P_) K^+ ^channels. These channels contribute to the regulation of membrane repolarization, resting membrane potential, frequency of firing, and excitability of sensory neurons [9,10]. Among various K^+ ^channels, K_v _channels play a crucial role in returning the depolarized cell to the resting state, and the inhibition of these channels leads to AP broadening (for review see [11]). In addition, DRG neurons express at least eight K_2P _channel subtypes as reported at the mRNA transcript level [12,13]. K_2P _channels help to set, and stabilize the resting membrane potential and closing of these channels leads to membrane depolarization [14]. In our preliminary analysis of the results, we found an increase in membrane resistance and duration of AP during IQ treatment. Therefore, we systematically tested the possible molecular target of IQ on K^+ ^channels. Our results demonstrate that IQ effectively inhibits K_v _and K_2P _channels in DRG neurons.

**Figure 1:**
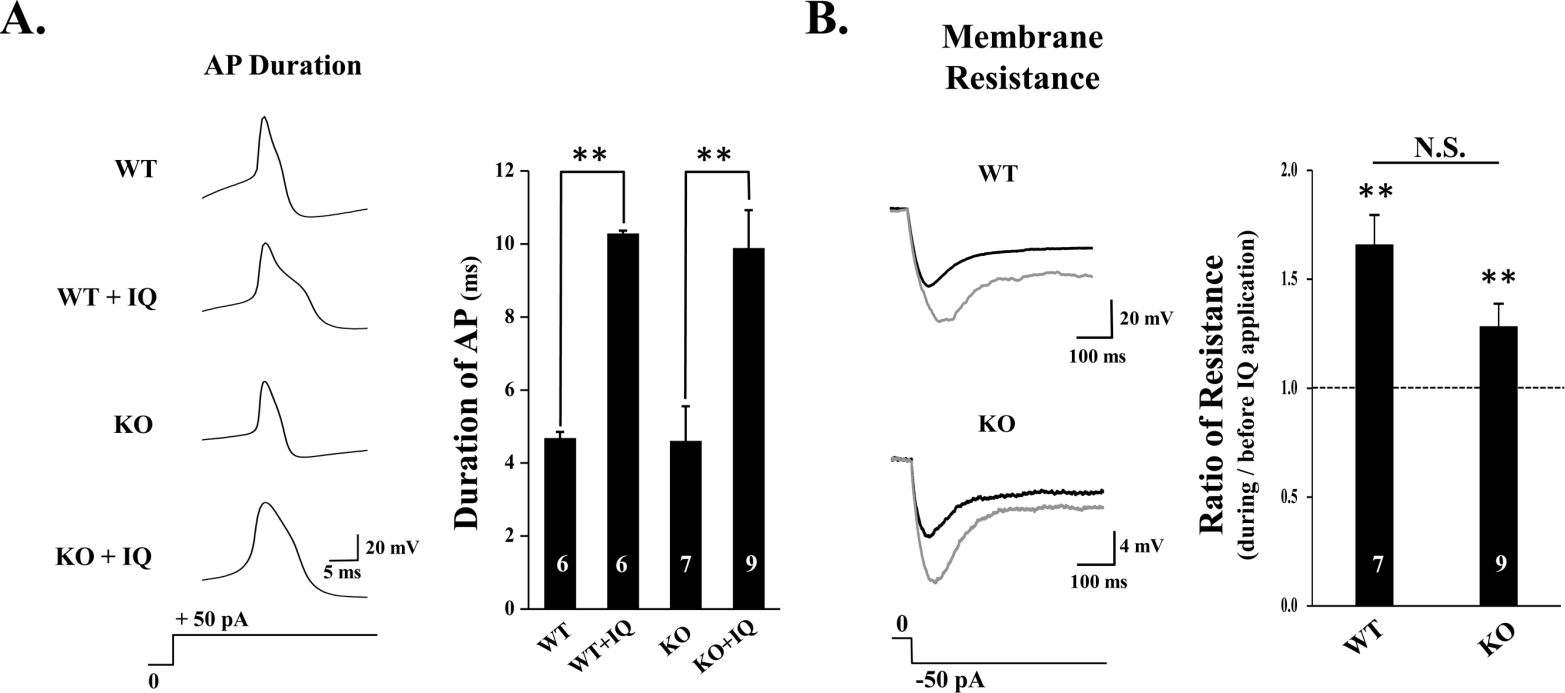
**Effects of IQ on AP duration and membrane resistance of DRG neurons in WT and TLR7 KO mice**. **A**. Representative trace of AP of DRG neuron from WT and TLR7 KO before and after application of IQ. Bar graph indicates the average of AP duration of DRG neurons with or without IQ treatment from WT and TLR7 KO mice. **B**. Left column shows the representative example of change of membrane resistance induced by injection of negative current. Average effect of IQ on membrane resistance compared with WT and KO is shown as bar graph. Protocol for AP induction and membrane resistance by current injection was shown in bottom of traces of A and C. The number in each bar indicates the number of neurons. Error bars indicate S.E.M. (**, P > 0.01).

Our previous results and initial examination of the action of IQ on AP waveform inspired us to closely monitor the changes of AP during the treatment of IQ. First we compared IQ evoked firing of AP on WT and TLR7 KO mice. Using whole cell recording under current clamp mode, we obtained action potential firing from DRG neuron induced by 50 pA current injection. Interestingly the half-width duration of AP from WT DRG neurons increased significantly by a twofold during the bath application of 20 μg/ml IQ (from 4.68 ± 0.53 ms (n = 10) to 10.28 ± 0.19 ms (n = 6), P < 0.01). Interestingly, this increase in AP duration by IQ was also observed in the DRG neurons of TLR7 KO (from 4.60 ± 0.95 ms (n = 7) to 9.89 ± 1.04 ms (n = 9), P < 0.01) (Figure 1A).

Then, we examined the change of input resistance by IQ in WT. The measurement of input resistance by the injection of negative current indicated that background leak channel activity was altered by IQ treatment (Figure 1B). Membrane resistance of WT and TLR7 KO was similar (593 ± 52.39 MΩ (n = 6) in WT control; 531 ± 151.99 MΩ (n = 6) in TLR7 KO) and IQ significantly increased membrane resistance in WT (165.66 ± 13.82% (n = 7), P < 0.01) and KO (128.11 ± % (n = 9), P < 0.05). These results indicated that IQ depolarizes the membrane potential and increase AP duration in DRG neurons, and that these effects might not be due to the activation of TLR7 signaling pathway. Rather, these results strongly suggested IQ's direct targeting on ion channel(s), in particular, potassium channels.

**Figure 2:**
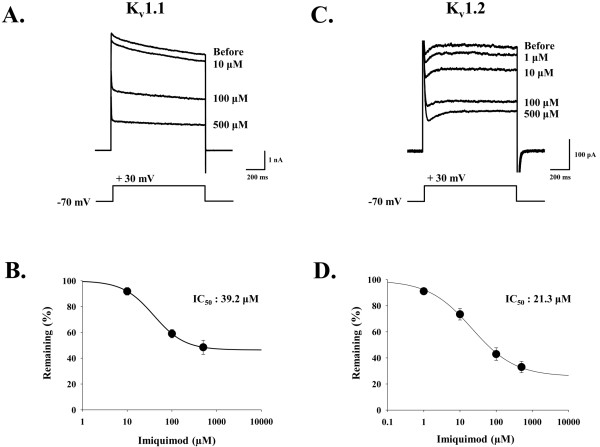
**Partial inhibitory effect of IQ on K_v_1.1 and K_v_1.2 activity**. **A, C **Effect of different concentrations of IQ on K_v_1.1 (A) and K_v_1.2 (C) currents induced by a voltage step. Protocol of voltage step shown below the traces. **B, D**. Average % of remaining currents of K_v_1.1 (B) and K_v_1.2 (D) during IQ (1, 10, 100 and 500 μM) application. Concentration-response relationship was obtained by fitting with logistic function. IC_50 _of IQ on K_v_1.1 and K_v_1.2 is 39.2 μM and 21.32 μM, respectively. Error bars indicate S.E.M.

K_v_1.1 and K_v_1.2 are the predominant voltage gated K^+ ^channels in DRG neuron that regulates the time course of AP waveform. Because we observed that IQ increased the duration of AP, we investigated whether IQ has any direct effect on the activity of K_v_1.1 and K_v_1.2 in heterologous expression system. Under whole-cell patch voltage clamp mode, inward and outward K^+ ^current through K_v_1.1 and K_v_1.2 channels were elicited by a series of voltage steps (from +100 mV to -100 mV with 20 mV step) to examine the effect of IQ on the voltage gated channels expressed in COS7 cells. The activity of both K_v_1.1 and K_v_1.2 were blocked by IQ treatment in a dose-dependent manner. K_v_1.1-mediated current was concentration-dependent. It was effectively inhibited by IQ at the concentrations of 10, 100 and 500 μM. The remaining currents were measured as 91.87 ± 2.94% (n = 9), 60.36 ± 3.26% (n = 11) and 48.49 ± 5.49% (n = 9), respectively (Figure 2A). Similarly K_v_1.2-mediated current also was dose-dependently blocked by IQ at 1, 10, 100, and 500 μM. The remaining currents were measured as 91.07 ± 2.87% (n = 4), 73.45 ± 5.43% (n = 7), 42.93 ± 5.48% (n = 7), and 33.01 ± 4.55% (n = 7), respectively (Figure 2B). The resulting concentration-response relationship showed that the efficacy of IQ on K_v_1.1 and K_v_1.2 is not completed at saturating concentrations, suggesting that IQ is acting as a partial antagonist. The half-maximal concentration, IC_50 _of IQ on K_v_1.1 and K_v_1.2 activity was determined as 39.2 μM (Hill Coefficient = 1.26) and 21.32 μM (Hill Coefficient = 0.7262) respectively.

**Figure 3:**
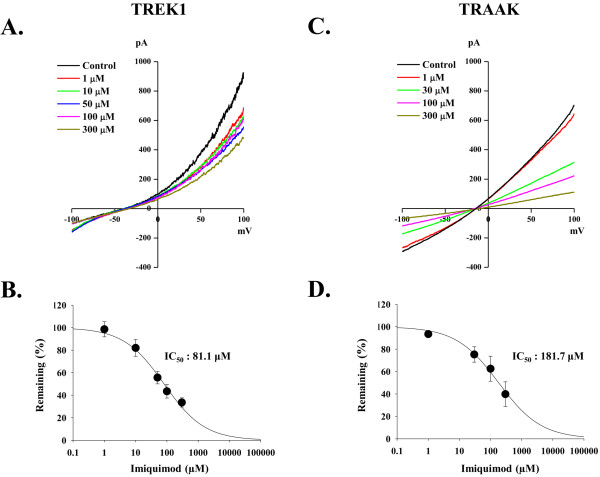
**Antagonistic effect of IQ on TREK1 and TRAAK, K_2P _channel**. **A, C **Inhibitory effect of IQ on TREK1 (A) and TRAAK (C) channels as shown by instant I-V relationship. **B, D **Average % of remaining currents at each concentration of IQ on TREK1 (B) and TRAAK (D) channels. Fitted line was used to obtain IC_50 _(TREK1, 81.1 μM; TRAAK, 181.7 μM) of IQ on K_2P _channels. Error bars indicate S.E.M.

To test IQ's effect on the leak current, we recorded the activity of heterologously expressed TREK1 and TRAAK, one of the major K_2P _channels expressed in DRG neurons. Whole cell currents elicited by voltage ramps from +100 mV to -100 mV for each concentration of IQ were recorded and analyzed for the degree of block. Figure 3A and 3C show the representative traces of TREK1 and TRAAK-mediated currents in different concentrations of IQ. The traces were induced by ramping membrane potential to obtain instant current-voltage relationship (see Materials and Methods). We found that IQ readily blocked TREK1 and TRAAK-mediated currents in a concentration-dependent manner (for TREK1, 1 μM: 1.5 ± 6.75%, 10 μM: 18.0 ± 7.40%, 50 μM: 44.3 ± 5.57%, 100 μM: 56.5 ± 5.99%, and 300 μM: 66.4 ± 4.07% (Figure 3B); for TRAAK, 1 μM: 6.6 ± 2.04%, 30 μM: 24.8 ± 6.85%, 100 μM: 37.5 ± 11.14%, and 300 μM: 60.2 ± 11.01% (Figure 3D)). The calculated IC_50 _of IQ on TREK1 and TRAAK currents was 181.7 μM and 81.1 μM after curve fitting. These results indicated that IQ directly blocks TREK1 and TRAAK channels, which provide the background leakage current in DRG neurons.

Clinically, IQ has been known as a topically active immunomodulatory agent that is formulated as a 5% cream for application by the patient of skin diseases. IQ 5% cream is generally well tolerated by immunocompetent and HIV-infected patients. Local skin reactions (mainly mild or moderate), including erythema, itching and burning, are the most commonly reported adverse events, occurring in < or = 67% of patients applying IQ 5% cream 3 times a week [1,5]. So far, the mechanism of how IQ causes the adverse side-effects has been unknown. In this study, we demonstrate, for the first time, that IQ inhibits K_v_1.1and K_v_1.2 and TREK1 and TRAAK. These results can explain the IQ's action of increasing the excitability of sensory neurons. This, in turn, could explain the ability of IQ to induce itching sensation.

In our previous study, we described various actions of IQ such as membrane depolarization, action potential firing, increase in intracellular Ca^2+ ^concentration, and itching behavior [7]. We found that IQ can directly excite DRG neurons most likely by blocking K_v_ and K_2P_ channels. For IQ-induced Ca^2+ ^response, we found that it was not altered by external Ca^2+^, but was completely blocked by the application of 2-aminoethoxydiphenyl borate (2-APB), an inhibitor of inositol trisphosphate receptor (IP3R) [7]. Therefore, IQ seems to act on many molecular targets including Ca^2+ ^release channels as well as various potassium channels in sensory neurons. Our data from the heterologous expression system using COS7 cells that lacks TLR7 re-confirm that IQ effects are TLR7-independent, and suggest that it rather directly acts on potassium channels in sensory neurons. In this study, we have not characterized the putative cross-talk between IQ-induced Ca^2+ ^increase and potassium channel block. Thus, at this point, it is not clear whether IQ inhibits potassium channels independently of intracellular Ca^2+ ^increase, which needs to be clarified in the future studies.

Although there are many channels contributing to membrane depolarization and generation of action potential in neurons, various K^+ ^channels exert an important role in repolarizing membrane potentials and maintaining AP duration. Among many types of potassium channels expressed in DRG neurons, K_v_1.1, K_v_1.2, K_vβ_2.1 and K_v_1.4 were reported as voltage-gated channels that regulate membrane repolarization, resting membrane potential, frequency of firing, and neurotransmitter release in sensory neurons [9]. Background K^+ ^channels have been implicated in various important physiological functions including mechano-, lipid-, acid- and possibly oxygen-sensing in specialized cells. In neurons, it was reported that these channels help to set and stabilize the resting membrane potential [14]. Our results demonstrate that IQ blocks K^+ ^channels (K_v _and K_2P_), thereby enhancing the excitability of DRG neurons. Our results provide a possible explanation for how IQ causes pruritius. Yet, the detailed mechanism of how IQ can lead to itching sensation by blocking various potassium channels remains to be uncovered.

## Materials and methods

### Animals

All experimental procedures described below were performed in accordance with the institutional guidelines of KIST (Seoul, Korea). TLR7-deficient mice were purchased from Jackson Laboratory. They were crossed with wild-type C57BL/6 (2-month-old, Charles River) to produce heterozygous mice. The resultant heterozygous mice were used as mating pairs. More detail methods maintaining TLR7 strain were previously described [7]. Specifically, the Jackson Laboratory TLR7 mouse was originally established by R. Flavell's laboratory (Yale University, New Haven, CT). Littermates or age-matched mice were generally used as wild type controls.

### Preparation of Primary DRG Cell Culture

DRG neurons were prepared from adult mice (~2 month old) by methods similar to those described previously [15,16]. Briefly, DRG from all spinal levels were collected in Ca^2+^/Mg^2+^-free Hanks' balanced salt solution (HBSS; Invitrogen) and treated with enzyme solution containing 60 units of papain/cystein (0.33 mg/mL), 5 mg/mL of Dispase, and 4 mg/mL of collagenase (Gibco) for 10 min at 37°C, respectively. Following trituration and centrifugation, the dispersed cells were resuspended in DMEM/F12 culture media containing 10% FBS [nerve-growth factor (NGF) or glial cell line-derived neurotropic factor (GDNF) was not included in culture medium] and were plated in 35-mm poly D-lysine precoated glass-bottomed dishes (MatTek) coated with laminin. Cultures were maintained at 37°C in a humidified atmosphere of 95% air and 5% CO_2 _and assayed after 16-20 h.

### DRG neuron whole cell recording

Acutely dissociated DRG neurons were obtained from C57BL/6 and TLR7 KO mice (8-10 wks) and attached to coverslips for recording. Before recording, coverslips were incubated in media for at least 1 h. DRG neuron recordings were performed in HEPES buffer of the following composition (in mM): 10 HEPES, 150 NaCl, 10 KCl, 2 CaCl_2_, 2 MgCl_2_, 5.5 glucose, and 22 sucrose (pH 7.4). Under the upright microscope, whole-cell patch recordings were obtained from acute-isolate DRG neurons in voltage-clamp mode and switched current-clamp configuration for recordings with an Axopatch 700B (Molecular Devices). Pipette resistance ranged from 3 to 6 MΩ. The internal solution consisted of (in mM) 140 K-gluconate, 10 Hepes, 7 NaCl, 4 Mg-ATP, and 0.3 Na_3_-GTP (pH 7.4). Firing properties were established for 1,000-ms duration by applying current injection from -100 to 350 pA with 50 pA increase. The cells with access resistance less than 20 MΩ that showed less than 20% change during the recordings were included in this study. Records were filtered at 5 kHz and digitized at 2-5 kHz with a Digidata 1322A (Molecular Devices) analog-to-digital board. Data were analyzed and plotted with the pClamp (Molecular Devices), Mini Analysis Program (Synaptosoft), and Origin. All data were collected at room temperature (23-26°C).

### COS7 Cell Culture and Transfection of plasmid DNA

COS 7 cells are cultured in high-glucose DMEM (Invitrogen) containing 10% FBS, and 100 U/ml penicillin/streptomycin, and maintained at 37°C in a 5% CO_2 _incubator. Transfections of plasmid DNA containing rat Kv1.1 (Genbank: NM_017303) and rat K_v_1.2 (Genbank: NM_012970) and mouse TREK1 (Genbank: NM_010607) and rat TRAAK (Genbank: NM_053804) were performed with Effectene transfection reagent (Qiagen). Protocols were conformed to the product guideline description. Condition of recording and internal solution was same with the condition of recording DRG neurons.

## Competing interests

The authors declare that they have no competing interests.

## Authors' contributions

All authors read and approved the final manuscript. JL, TK, JH and JW conducted the majority of the experiments and analyzed data. HM conducted the preparation of DRG neurons. JL, EH, SJL and CJL designed experiments. JL and CJL contributed to all the experiments and data analysis and writing the manuscript
